# Analyzing small RNA sequences from canine stem cell-derived extracellular vesicles primed with TNF-α and IFN-γ and exploring their potential in lung repair

**DOI:** 10.3389/fvets.2024.1411886

**Published:** 2024-07-01

**Authors:** Ji-Sun Lee, Yun-Ho Jeong, Yo-Han Kim, Jang-Hyuk Yun, Jin-Ok Ahn, Jin-Young Chung, Ju-Hyun An

**Affiliations:** ^1^Department of Veterinary Emergency and Critical Care Medicine, College of Veterinary Medicine, Kangwon National University, Chuncheon, Republic of Korea; ^2^Department of Veterinary Internal Medicine, College of Veterinary Medicine, Institute of Veterinary Science, Kangwon National University, Chuncheon, Republic of Korea; ^3^Department of Large Animal Internal Medicine, College of Veterinary Medicine, Kangwon National University, Chuncheon, Republic of Korea; ^4^Department of Veterinary Pharmacology, College of Veterinary Medicine, Institute of Veterinary Science, Kangwon National University, Chuncheon, Republic of Korea

**Keywords:** acute lung injury, canine, endothelial to mesenchymal transition, extracellular vesicle, stem cell

## Abstract

Acute lung injury is an acute inflammation disorder that disrupts the lung endothelial and epithelial barriers. In this study, we investigated the extracellular vesicles (EVs) obtained via priming inflammatory cytokines such as tumor necrosis factor (TNF)-α and interferon (IFN)-γ on canine adipose mesenchymal stem cells in improving their anti-inflammatory and/or immunosuppressive potential, and/or their ability to alleviate lipopolysaccharide-induced lung injury *in vitro*. We also explored the correlation between epithelial-to-mesenchymal transition and the inflammatory repressive effect of primed EVs. Using small RNA-Seq, we confirmed that miR-16 and miR-502 significantly increased in EVs from TNF-α and IFN-γ-primed canine adipose mesenchymal stem cells. The pro and anti-inflammatory cytokines were analyzed in a lipopolysaccharide-induced lung injury model and we found that the EV anti-inflammatory effect improved on priming with inflammatory cytokines. EVs obtained from primed stem cells effectively suppress endothelial-to-mesenchymal transition in a lung injury model. Our results suggest a potential therapeutic approach utilizing EVs obtained from adipose mesenchymal stem cells primed with TNF-α and IFN-γ against lung inflammation and endothelial to mesenchymal transition.

## 1 Introduction

Acute lung injury (ALI) is defined by hypoxemic respiratory insufficiency due to non-cardiogenic pulmonary edema resulting from increased pulmonary vascular permeability (1). Despite the reported mortality rate of over 90% in veterinary medicine, there is still a lack of specific and effective treatment methods for ALI (2).

Lung endothelial cells form the pulmonary vascular bed and constitute the majority of cells in the lungs. During ALI, systemic inflammation affects the pulmonary endothelium, compromising the integrity of the alveolar capillary membrane and leading to the accumulation of edematous fluid in the lung parenchyma. Consequently, this cascade results in ventilation failure (3, 4). In addition to their role in gas exchange, lung endothelial cells create a specialized microenvironment, or niche, with significant implications for both health and disease. Therefore, the notion that the restoration of lung endothelial cells (ECs) is crucial for ALI treatment has garnered considerable attention.

Recent studies have highlighted a critical therapeutic role for extracellular vesicles (EV) secreted by mesenchymal stem cells (MSCs) in ALI (5, 6). EVs are nano-sized membrane-bound vesicles that transport essential biomolecules between cells to maintain physiological homeostasis. Research indicates that EVs derived from MSCs (MSC-EV) possess regenerative and anti-inflammatory properties (7, 8). Utilizing MSC-EVs as an alternative to MSCs offers several advantages, including a higher safety profile, lower immunogenicity, and the ability to traverse biological barriers. Moreover, ongoing studies aim to enhance the efficacy of stem cell-derived extracellular vesicles. Among these efforts, the anti-inflammatory efficacy of MSC-EVs has been augmented by priming stem cells with interferon (IFN)-γ and tumor necrosis factor (TNF)-α (9, 10). However, further research is necessary to ascertain the impact of applying MSC-EVs with enhanced therapeutic properties to lung diseases.

We hypothesized that primed EVs may enhance therapeutic effectiveness in repairing lung endothelial cells. To investigate this, we first investigated factors associated with tissue recovery by profiling miRNAs in canine MSC-EVs obtained after priming TNF-α and IFN-γ. We also used *in vitro* lung injury model, a lipopolysaccharide (LPS)-induced lung endothelial cell, to compare and analyze the degree of inflammation alleviation provided by primed and naive canine MSC-EVs.

## 2 Materials and methods

### 2.1 The Institutional Animal Care and Use Committee

This study was conducted following the protocols approved by the Institutional Animal Care and Use Committee of Kangwon National University (KNU), Republic of Korea, and in compliance with the authorized guidelines (KNU, Protocol No. KW-230912-3).

### 2.2 Isolating TNF-α and IFN-γ-primed canine adipose mesenchymal stem cell EVs

Stem cells were isolated from canine adipose tissue from 3 dogs and primed with inflammatory cytokines (TNF-α and IFN-γ) as previously described (9, 11). To summarize briefly, the canine adipose-derived mesenchymal stem cells (cASCs) were seeded in 6-well plates at a density of 5 × 10^5^ cells per well and cultured in Dulbecco’s modified Eagle’s medium with 4.5 g/L glucose (DMEM; PAN-Biotech, Aidenbach, Germany) supplemented with 10% Exo-free fetal bovine serum (FBS; PAN-Biotech) and 1% penicillin-streptomycin (PS; PAN-Biotech). After 6 h, the cells were stimulated for 24 h with TNF-α (20 ng/mL, PROSPEC, Ness Ziona, Israel) and IFN-γ (20 ng/mL, Kingfisher Biotech, Saint Paul, MN). Then, the conditioned media in the 6-well plate was discarded and washed three times with DPBS (PAN-Biotech). To isolate EVs, cASCs were cultured for 48 h in Dulbecco’s modified Eagle’s medium (DMEM) supplemented with 5% exosome-depleted fetal bovine serum (FBS; Thermo Fisher Scientific, MA, United States). Following incubation, the medium from each cultured cASC sample was harvested and centrifuged at 100 × g for 5 min. Then, to exclude cells and cellular debris, each supernatant was transferred to a fresh tube and ExoQuick-CG Exosome Precipitation Solution (System Biosciences, CA, United States) was added at a 3:1 ratio of supernatant to ExoQuick-CG Exosome Precipitation Solution. After incubation in the refrigerator for 12 h, the ExoQuick-CG/supernatant mixture was centrifuged at 1,500 × g for 30 min. After centrifugation, exosomes were identified as white pellets, and these exosomes were used in experiments. Exosome characterization was conducted in a previous study (10). Briefly, protein markers of isolated EVs were detected using western blotting with antibodies targeting CD81 (Aviva system biology, CA, United States) and CD9 (GeneTex, Irvine, CA, United States). The morphology of the EVs was assessed using transmission electron microscopy. Briefly, 10 μL of EV suspension was applied onto a 300-mesh formvar/carbon-coated electron microscopy grid with the coated side facing the suspension. The grid was washed with distilled water and then negatively stained with a 10 μL drop of uranyl acetate for 1 min. The EVs were observed under a transmission electron microscope (TEM; LIBRA 120, Carl Zeiss, Germany) operating at 120 kV. The size distribution of the particles was determined using a zeta-potential and particle size analyzer (ELSZ-1000ZS, Otsuka Electronics, Osaka, Japan). Total protein concentration of EVs was determined using bicinchoninic acid (BCA) assays (Bio-Rad lab, CA, United States).

### 2.3 Identifying miRNAs via small-RNA sequencing analysis

Small RNA sequencing assay was conducted by E-Biogen, Inc. (Seoul, Republic of Korea). Libraries for both Naive and primed EV RNAs were prepared using the NEBNext Multiplex Small RNA Library Prep kit (New England BioLabs, Inc., MA, United States) as per manufacturer’s instructions. Initially, the total RNA from each sample was used for adaptor ligation, followed by complementary DNA (cDNA) synthesis using reverse transcriptase with adaptor-specific primers. Subsequently, polymerase chain reaction (PCR) amplification was performed to generate libraries, which were purified using the QIAquick PCR Purification Kit (Qiagen, Hilden, Germany) and subjected to polyacrylamide gel electrophoresis. The yield and size distribution of the small RNA libraries were assessed using an Agilent 2100 Bioanalyzer instrument with a High-sensitivity DNA Assay (Agilent Technologies, Inc., CA, United States). High-throughput sequencing was performed on a NextSeq550 system, employing single-end 75 sequencing (Illumina Inc., CA, United States). Sequence reads were aligned using the bowtie2 software tool to generate a BAM file with mature miRNA sequences serving as references for mapping. For determination of RNAseq, the software programs bedtools v2.25.0 (Quinlan and BEDTools), mirbase.db v1.2.0 and microRNA v1.58.0 in Bioconductor (12) were employed in conjunction with R statistical programming language (13) to map read counts onto mature miRNA sequences and extract them from the alignment file.

### 2.4 Canine lung-derived endothelial cell isolation

Stromal cells were isolated from the lung tissues obtained from three dogs. Tissue samples were obtained from one biopsy per dog and washed twice with Dulbecco’s phosphate-buffered saline (DPBS; PAN-Biotech GmbH, Aidenbach, Germany) containing 1% penicillin-streptomycin (PS; PAN-Biotech). The washed tissues were plated on a Petri dish, digested with 10 mL of 0.1% Collagenase type 1A solution (Gibco/Life Technologies, MA, United States), and incubated for 50 min at 37°C in a humidified atmosphere with 5% carbon dioxide. Following digestion, DMEM (PAN-Biotech) supplemented with 10% FBS; (Sigma-Aldrich, MO, United States) and 1% PS was added to neutralize the collagenase. The suspension was centrifuged at 1,200 × g for 5 min, the supernatant was discarded, and the cell pellet was resuspended in DMEM. The suspension was then passed through a 70 μm Falcon cell strainer (Thermo Fisher Scientific) to remove debris, followed by centrifugation at 1,200 × g for 5 min. To remove erythrocytes, red blood cell lysis buffer (Sigma-Aldrich) was added and the cell suspension was incubated for 10 min at 25°C. The cells were washed with DPBS and the supernatant was discarded. The cells were then resuspended in DMEM.

Then, the cells were incubated with a canine CD31 antibody (dilution, 1:200; Invitrogen, CA, United States) for 1 h at 25°C, followed by centrifugation at 1,200 × g for 5 min. Subsequently, the cells were incubated with anti-mouse immunoglobulin (Ig)-G microbeads (Miltenyi Biotec, CA, United States) for 1 h at 25°C, followed by magnetic-activated cell sorting (MACS; Miltenyi Biotec) to isolate CD31^+^ cells according to the manufacturer’s instructions. PBS with 10% FBS (PAN-Biotech) was used as the MACS separation buffer. The lung stromal cells were incubated with the CD31 antibody for 2 h at 37°C in a humidified atmosphere with 5% carbon dioxide. CD31^+^ endothelial cells were collected after centrifugation at 780 × g for 10 min. Magnetic labeling was performed via adding 10 μL of anti-mouse IgG microbeads and 40 μL MACS separation buffer to the CD31^+^ cells, followed incubation for 2 h. The CD31^+^ cells were collected after centrifugation at 780 × g for 10 min. After washing the MACS LS column with 3 mL MACS separation buffer, the CD31^+^ endothelial cells were added to it for isolation. After another wash with 3 mL MACS separation buffer, the cells were collected in 5 mL MACS separation buffer.

### 2.5 LPS-induced lung inflammation *in vitro*

To stimulate endothelial cells with LPS (Sigma-Aldrich), the cells were seeded at a density of 1 × 10^5^ cells/mL in six-well plates. A control medium, devoid of LPS, was used alongside the experimental groups to which 200 ng/mL LPS was added. Following 24 h of incubation for stimulation, the medium was replaced with media containing EVs derived from naive (unstimulated) or primed (stimulated) cASCs at a concentration of 50 μg/mL (11). Subsequently, the cells were further incubated for 48 h before harvesting for RNA extraction and enzyme-linked immunosorbent assay (ELISA) analysis.

### 2.6 Cell proliferation assay

To examine the potential impact of LPS and EVs on canine lung endothelial cell proliferation, it was evaluated using a Cell Counting Kit-8 (CCK-8) assay (D-PlusTM CCK Cell Viability Assay Kit; Dong-In Biotech, Seoul, Republic of Korea). Endothelial cells were seeded at a density of 3.3 × 10^4^ cells/well in 96-well plates. Following co-culture with LPS (200 ng/mL) and EVs (50 μg/mL), a CCK-8 assay was performed at either 24 or 48 h.

### 2.7 Evaluation of gene expression

RNA was extracted from lung endothelial cells at a density of 2.5 × 10^5^ cells per well using the Easy-Blue Total RNA Extraction Kit (Intron Biotechnology, Sungnam, Republic of Korea) following the manufacturer’s instructions. Subsequently, cDNA was synthesized using the Cell Script All-in-One 5x 1st cDNA Strand Synthesis Master Mix (Cell Safe, Seoul, Korea) containing M-MLV (Moloney Murine Leukemia Virus) reverse transcriptase (RTase), ribonuclease inhibitor, dNTPs and an optimized ratio of Oligo (dT)s and random primers. The synthesized cDNA samples were analyzed using the AMPIGENE qPCR Green Mix Hi-ROX with SYBR Green dye (Enzo Life Sciences, NY, United States) and forward and reverse primers (Bionics, Seoul, Republic of Korea). For relative quantification we used the 2^−ΔΔCT^ method (14). The target gene expression levels were normalized to those of glyceraldehyde 3-phosphate dehydrogenase (GAPDH). Primers utilized in this study targeted GAPDH, interleukin (IL)-1β, TNF-α, IL-10, N-cadherin, Vimentin, and E-cadherin present in dogs, with sequences detailed in Table 1.

**Table 1:** Sequences of PCR primers used in this study.

Gene	Forward (5′–3′)	Reverse (5′–3′)
Canine GAPDH	CCA TCT TCC AGG AGC GAG AT	TTC TCC ATG GTG GTG AAG AC
Canine IL10	CGA CCC AGA CAT CAA GAA CC	CAC AGG GAA GAA ATC GGT GA
Canine TNFA	CGT CCA TTC TTG CCC AAA C	AGC CCT GAG CCC TTA ATT C
Canine IL1Β	CCC TGG AAA TGT GAA GTG CT	CTG ACA CGA AAT GCC TCA GA
Canine ECAD	AAA ACC CAC AGC CTC ATG TC	CAC CTG GTC CTT GTT CTG GT
Canine NCAD	CCC AAG ACA AGC GAC TAA GC	TGA CAG CTG ACC TGA GAT GG
Canine VIMENTIN	CCG ACA GGA TGT TGA CAA TG	TCA GAG AGG TCG GCA AAC TT

### 2.8 ELISA

To investigate the secretion of TGF-β1 by lung endothelial cells in lung inflammation alongside canine stem cell EVs, the canine TGF-β1 concentration in the endothelial cell culture supernatant was measured using a TGF-β1 ELISA Kit (Novus Biologicals LLC, CO, United States), as per manufacturer’s instructions.

### 2.9 Statistical analysis

Statistical analyses were conducted using GraphPad Prism version 6.01 software (GraphPad Software, CA, United States). Student’s *t*-test and one-way analysis of variance (ANOVA) were used to analyze the data, followed by the Bonferroni multiple comparison test for post-hoc analysis. Results are expressed as mean ± standard deviation. Statistical significance was defined as *p* < 0.05. For RNAseq, the software programs bedtools v2.25.0 (Quinlan and BEDTools) and Bioconductor (12) were employed in conjunction with R statistical programming language (13) to map read counts onto mature miRNA sequences and extract them from the alignment file. These read counts were then used to assess miRNA expression levels using the CPM + TMM normalization method for inter-sample comparison.

## 3 Results

### 3.1 Comparing miRNA profiling between naive and primed cASC-derived EVs

**Figure 1:**
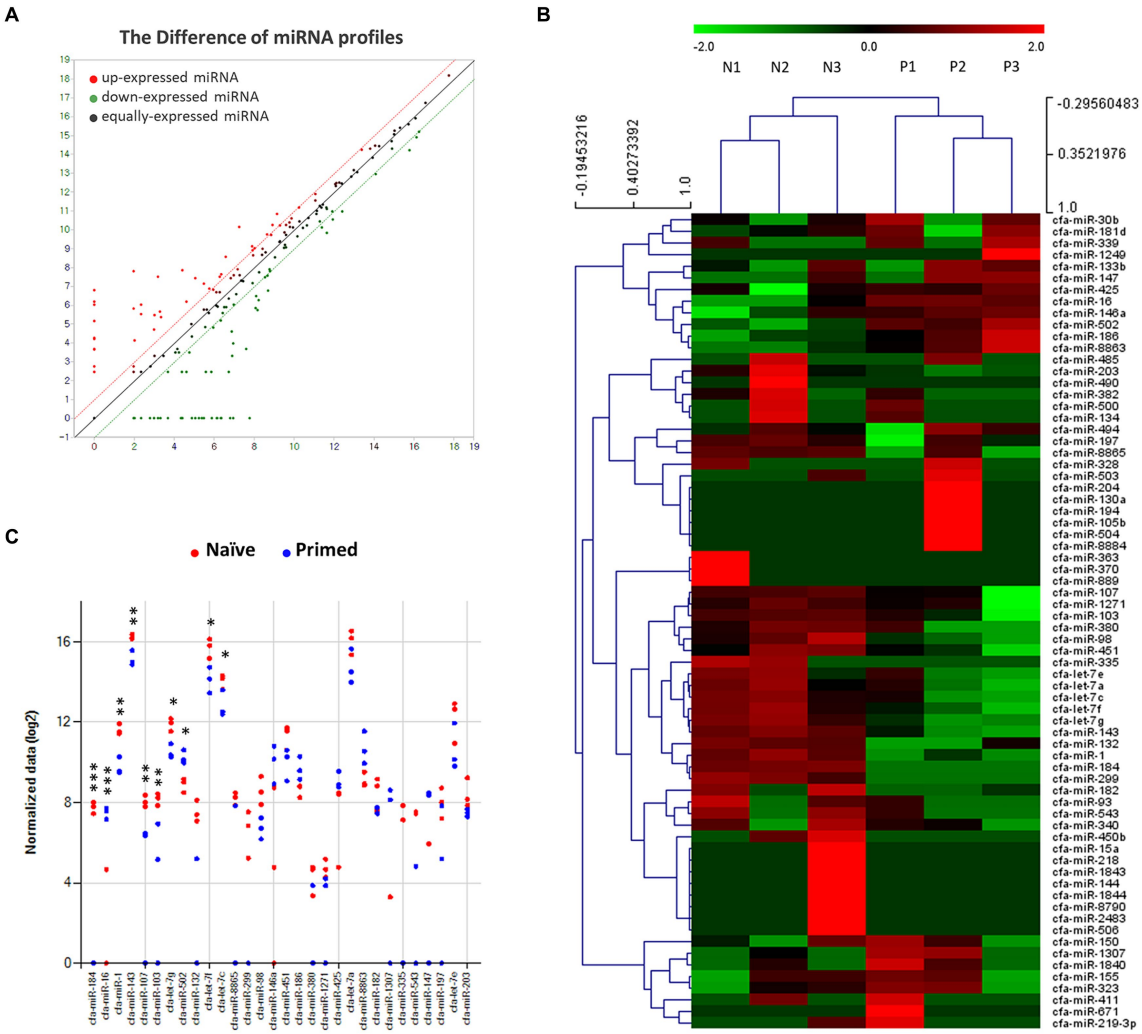
Profiles of miRNA expression in extracellular vesicles form naive and tumor necrosis factor (TNF)-α and interferon (IFN)-γ-primed canine stem cells. **(A)** Biological replicates between extracellular vesicles from naïve (*n* = 3) and TNF-α and IFN-γ-primed canine stem cells (*n* = 3). **(B)** Heatmap diagram of differential miRNA expressions between extracellular vesicles derived from naïve (*n* = 3) and TNF-α and IFN-γ-primed canine stem cells (*n* = 3). Mean expression values are shown. Red, increased expression; green, decreased expression; black, mean value. **(C)** Significant chart of miRNA expression in extracellular vesicles form naive (*n* = 3) and TNF-α and IFN-γ-primed canine stem cells (*n* = 3). (^*^p < 0.05, ^**^p < 0.01, and ^***^p < 0.001).

Differential miRNA expression patterns were observed in EV samples derived from both naive and primed cASCs. The expression level of each gene is reported as the read count normalized to the log_2_ value. Out of 455 total dog miRNAs in miRbase^1^ at the time of the analysis, 202 were dysregulated. Specifically, 95 miRNAs were upregulated, whereas 107 miRNAs were downregulated in primed cASC-derived EVs compared to their naive counterparts. A total of 11 miRNAs exhibited significant differences (*p* < 0.05) between naive and primed cASC-derived EVs (Figure 1).

### 3.2 Elevated miR-16 and miR-502 in primed cASC-derived EVs

**Figure 2:**
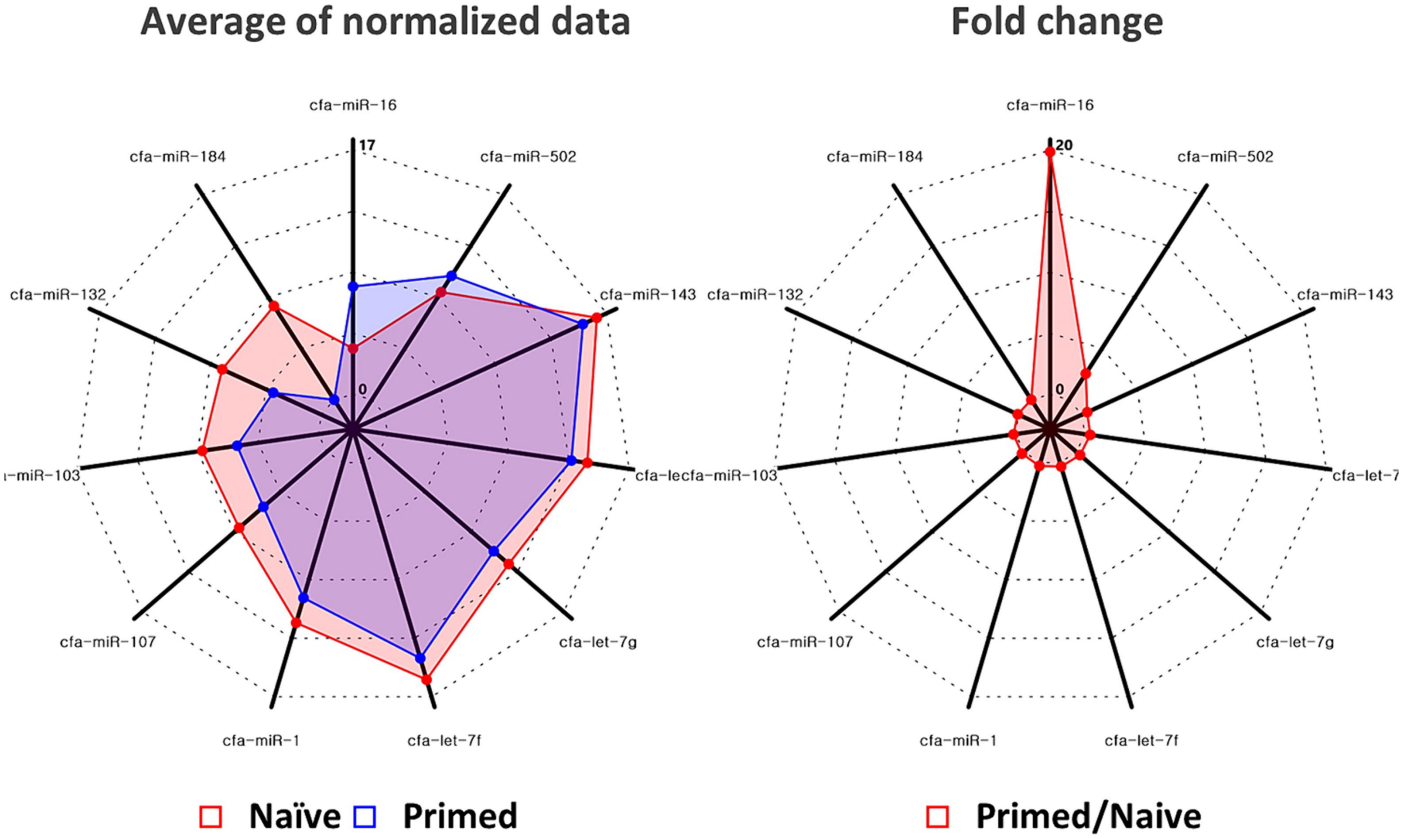
Quantitative real-time polymerase chain reaction validated the increase of miR-16 and miR-502 in extracellular vesicles derived from naïve (*n* = 3) and tumor necrosis factor (TNF)-α and interferon (IFN)-γ-primed canine stem cells (*n* = 3). Among miRNAs in which significant changes were confirmed, significant elevations were confirmed for miR-16 and miR-502.

In primed cASC-derived EVs, 11 miRNAs were significantly differentially expressed (*p* < 0.05, fold change >2.0). Among these, two miRNAs, miR-16 and miR-502, were upregulated, whereas the remaining nine miRNAs, miR-184, miR-132, miR-103, miR-107, miR-1, let-7f, let-7g, let-7c, and miR-143, were downregulated (Figure 2).

### 3.3 Proliferation of lung endothelial cells under naive and primed cASC-derived EVs

**Figure 3:**
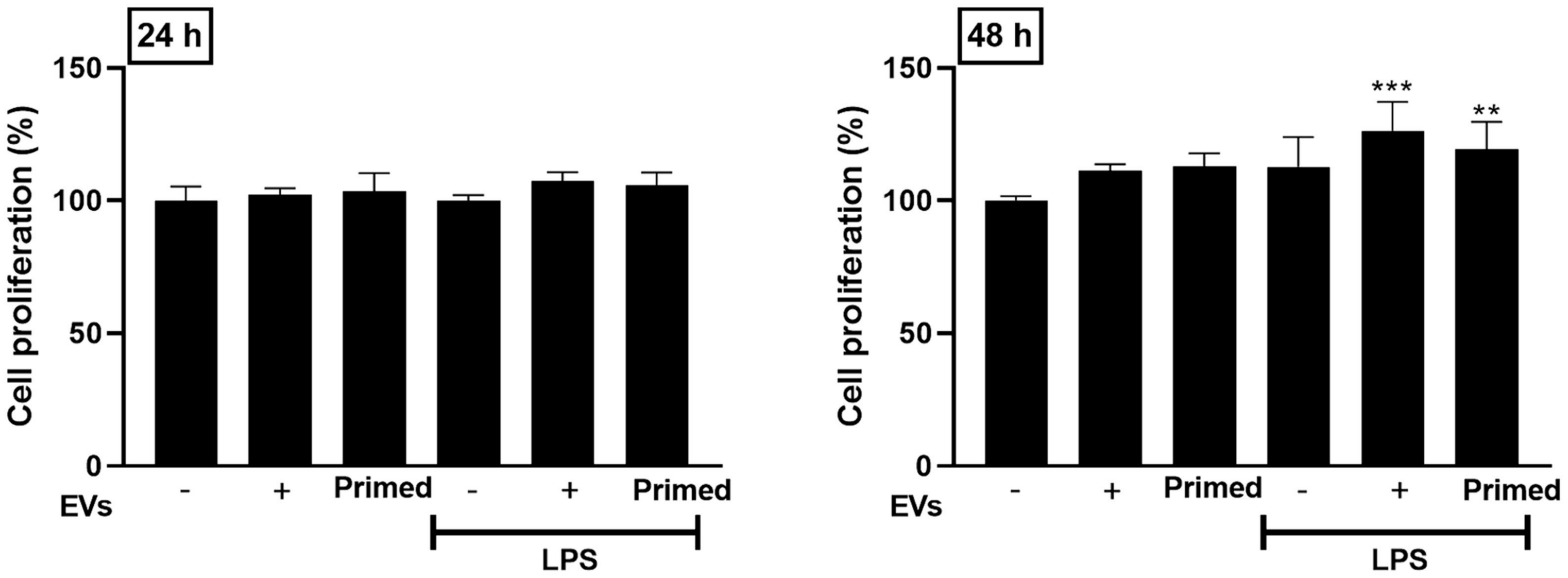
Effects of primed extracellular vesicles on lung endothelial cell proliferation *in vitro*. Cell proliferation was confirmed to increase when extracellular vesicles were used in the lung injury model, but no significant differences between the two extracellular vesicle groups were confirmed. The results are presented as the mean ± SD of triplicate samples and are representative of 3 independent experiments. (^**^p < 0.01 and ^***^p < 0.001).

The potential impact of LPS and EVs on canine lung endothelial cell proliferation were investigated using the CCK-8 assay, and significant cell proliferation was confirmed in endothelial cells treated with naïve and primed EV after 48 h (*p* < 0.01). However, no significant differences were observed between the two groups (Figure 3).

### 3.4 TNF-α and IFN-γ-primed EVs derived from cASCs for mitigating lung inflammation

**Figure 4:**
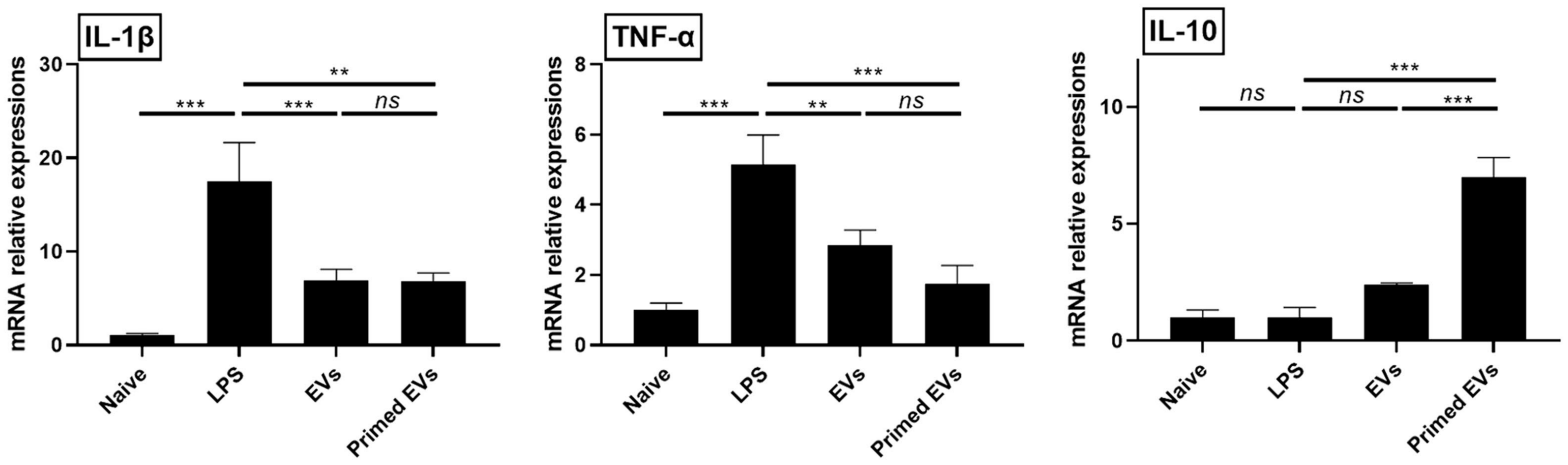
Immunomodulatory effects of extracellular vesicles from tumor necrosis factor (TNF)-α and interferon (IFN)-γ-primed canine stem cells in a lipopolysaccharide (LPS)-induced lung injury model *in vitro*. Changes in pro- and anti-inflammatory cytokines in LPS-activated lung endothelial cells. The results are presented as the mean ± SD of triplicate samples and are representative of 3 independent experiments.^*^p < 0.05, ^**^p < 0.01, ^***^p < 0.001, and ^****^p < 0.0001; ns, not significant.

To evaluate the immunomodulatory potential of primed cASC-derived EVs in lung inflammation, we analyzed the mRNA expression of anti- and proinflammatory cytokines in lung endothelial cells. Following LPS stimulation, TNF-α and IL-1β showed elevated expression levels compared to unstimulated lung endothelial cells (*p* < 0.001). However, when LPS-stimulated lung endothelial cells were cultured with naive and primed cASC-derived EVs, the TNF-α and IL-1β expression levels reduced (*p* < 0.01). We also assessed the mRNA expression of IL-10 and they were significantly elevated in LPS-stimulated lung endothelial cells cultured with primed cASC-derived EVs compared to those cultured with naive cASC-derived EVs (*p* < 0.001). This suggests that primed cASC-derived EVs may possess enhanced immunomodulatory properties, as evidenced by the upregulation of IL-10 expression (Figure 4).

### 3.5 TGF-β1 levels in lung endothelial cell-cultured media

**Figure 5:**
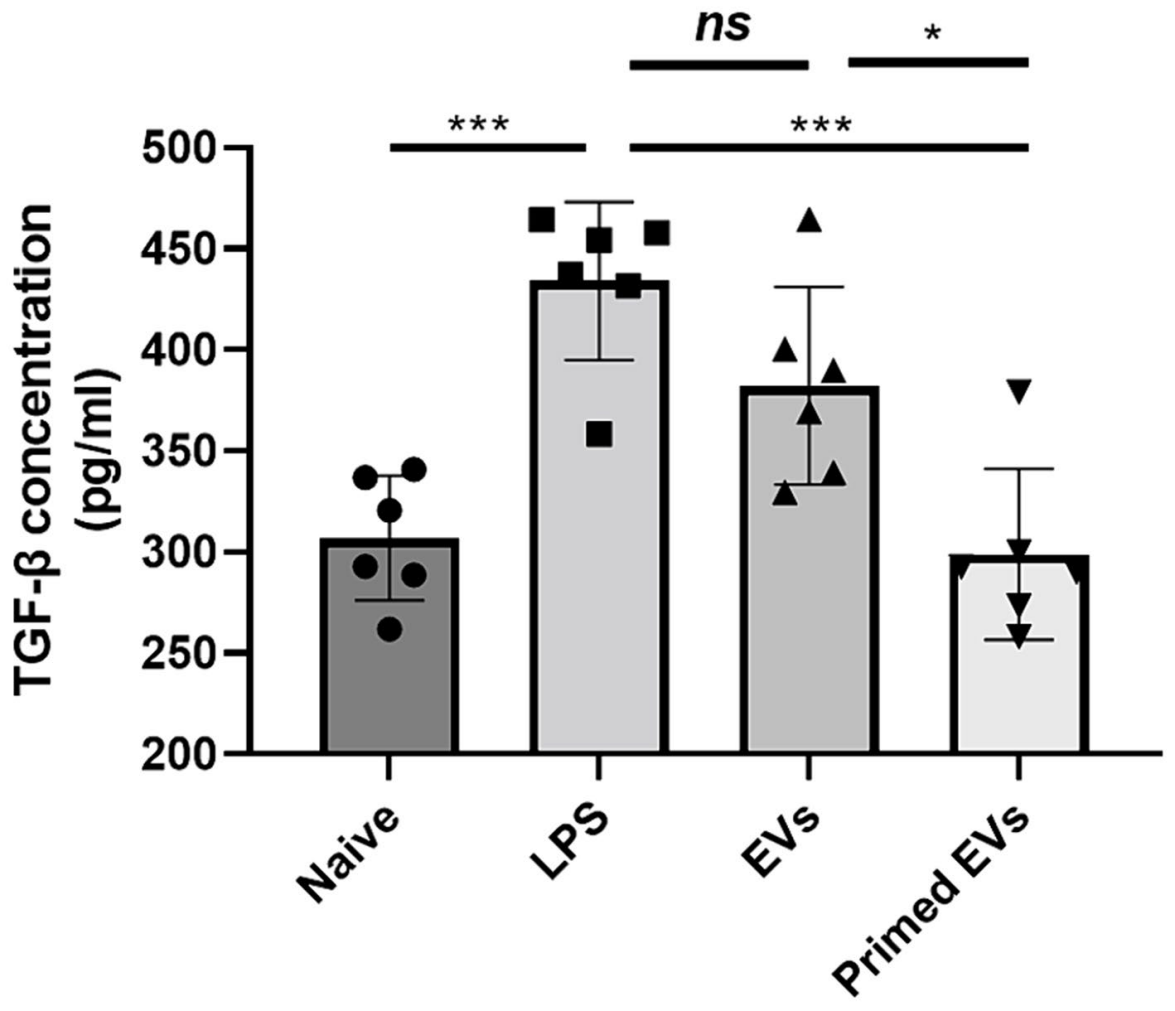
Transforming growth factor (TGF)-β1 expression in conditioned medium in lipopolysaccharide (LPS)-induced lung injury *in vitro*. In extracellular vesicles derived from canine stem cells primed with tumor necrosis factor (TNF)-α and interferon (IFN)-γ, the ability to reduce TGF- β1 was confirmed to have improved. The results are presented as the mean ± SD of triplicate samples and are representative of 3 independent experiments. ^*^p < 0.05, ^**^p < 0.01, ^***^p < 0.001, and ^****^p < 0.0001; ns, not significant.

The TGF-β1 ELISA Kit was utilized to quantify TGF-β1 levels in endothelial cell culture supernatant, either treated or not treated with naive and primed cASC-derived EVs. Among LPS treated groups, reduced TGF-β1 levels were observed in the conditioned media treated with both naive and primed cASC-derived EVs compared to the untreated cASC conditioned media. Furthermore, in the group treated with primed cASC-derived EVs, a significant decrease was observed compared to that in the group treated with naive cASC-derived EVs (*p* < 0.05) (Figure 5).

### 3.6 Inhibiting endothelial to mesenchymal transition in inflamed lung endothelial cells *in vitro*

**Figure 6:**
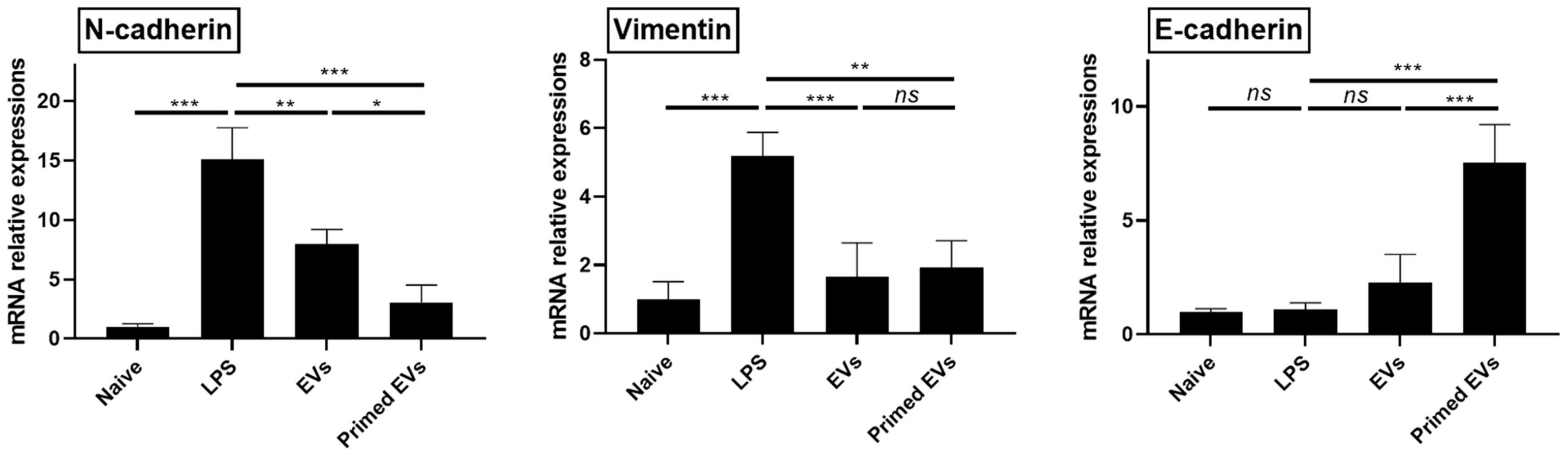
Expression of endothelial to mesenchymal transition marker in a lipopolysaccharide (LPS)-activated lung endothelial cell conditioned by extracellular vesicles derived from canine stem cells primed with tumor necrosis factor (TNF)-α and interferon (IFN)-γ. The results are presented as the mean ± SD of triplicate samples and are representative of 3 independent experiments. ^*^p < 0.05, ^**^p < 0.01, ^***^p < 0.001, and ^****^p < 0.0001; ns, not significant.

Upon exposure to LPS, lung endothelial cells exhibited increased expression levels of N-cadherin and Vimentin compared to their unstimulated counterparts (*p* < 0.001). However, co-culturing LPS-stimulated lung endothelial cells with naive and primed EVs derived from cASCs resulted in diminished expression of N-cadherin and Vimentin (*p* < 0.01). In addition, the E-cadherin mRNA expression levels significantly increased in LPS-stimulated lung endothelial cells cultured with primed cASC-derived EVs in contrast to those cultured with naive cASC-derived EVs (*p* < 0.001). This observation implied that primed cASC-derived EVs may impede the endothelial-to-mesenchymal transition during lung inflammation (Figure 6).

## 4 Discussion

In this study, we identified therapeutic efficacy factors of inflammatory cytokine-primed stem cell-derived EVs using RNAseq. In addition, the mechanism of lung recovery was confirmed by culturing primed EVs with lung endothelial cells with LPS-induced inflammation and analyzing EMT markers and inflammatory cytokines in the endothelial cells.

The pulmonary endothelium forms a continuous monolayer of squamous cells that line the internal surface of blood vessels (15). Together with the collagen-rich basement membrane, it serves to separate tissue microenvironments while also selectively connecting them to the bloodstream, thus contributing to tissue homeostasis (16). Previous studies have shown that acute or severe lung injury can lead to dense epithelial remodeling, resulting in hypoxic vasoconstriction and cytokine-induced inflammation in regions of severe damage. Additionally, it has been reported that recovery from lung endothelial cell injury is a key treatment goal for ALI (17). In our study, we confirmed the potential of primed EVs to promote lung repair using an *in vitro* model of LPS-induced inflamed lung endothelial cells. As a result, we observed a decrease in the expression of pro-inflammatory cytokines and an increase in the expression of anti-inflammatory cytokines, indicating the ability of primed EVs to alleviate inflammation and promote lung recovery.

TNF-α and IL-1β are the most commonly studied cytokines in acute inflammation studies because of their proinflammatory effects, including leukocyte chemoattraction and phagocytosis stimulation (18). An imbalance between pro and anti-inflammatory cytokines is related to an increased mortality rate in patients with acute respiratory distress syndrome (19). TNF-α and IL-1β have synergy during inflammation (20); hence, some studies have investigated the combined blockade of these cytokines simultaneously in imbalanced situations to control inflammation (18). Otherwise, IL-10 could suppress TNF-α production, inhibiting neutrophil activity and hence decreasing lung tissue damage (21). We found that both proinflammatory cytokines were expressed at lower levels in a lung inflammation co-cultured with EVs. In addition, IL-10 expression significantly increased in inflammatory lung cells co-cultured with primed cASCs. Previous studies have found that stem cells can produce immunomodulatory cytokines such as IL-10 to inhibit proinflammatory cytokine secretions such as TNF-α and IL-1β, thereby alleviating pulmonary inflammation (22, 23). The ability of EVs demonstrated similar results, and our study further suggests a treatment strategy targeting cytokine balance in canine pulmonary injuries.

TGF-β1 is correlated to macrophage and fibroblast recruitment (24), apoptosis in alveolar cells after damage (25), reactive oxygen species production (26) and EMT type II initiation (27). Ideally, the TGF-β1 activation leads to tissue repair and adequate regeneration. However, highly activated TGF-β1 can harmfully affect tissues and cause fibrosis and scarring after healing. Therefore, targeting TGF-β1 after lung injury could result in successful treatment with minimal side effects. In this study, we found that primed EVs were more effective than naive EVs in reducing TGF-β1 levels. Therefore, this is a significant finding that may assist in further studies on potential therapies.

We demonstrated that primed EVs derived from cASCs were more effective than naive EVs in inhibiting EMT *in vitro*. Both primed EVs could more effectively control EMT progression than naive EVs, which is considered to occur after inflammatory stimuli are identified by the increased E-cadherin expression. E-cadherin is an epithelial protein that is downregulated during disassembly of epithelial cell junctions (28, 29). In addition, regardless of priming, EVs reduced the expression of N-cadherin and Vimentin, which are mesenchymal markers found in EMT progression (27). EMT is a key factor in lung fibrosis and tissue remodeling that results from chronic respiratory disease; inhibition of EMT has been proved to attenuate fibrosis (30–32). Although further research is required prior to its application *in vivo*, this finding is useful in developing treatments for chronic respiratory diseases.

EVs obtained from primed MSC have been reported to have higher immunoregulatory functions than EVs obtained from naïve MSC (33, 34). Similarly, mesenchymal stem cells primed with inflammatory cytokines are known to exhibit enhanced immune-regulating effects (35). As indicated by our results, EVs primed with inflammatory cytokines exhibit superior immunoregulatory results. The miRNA profile analysis of naive and primed cASC-derived EVs exhibited distinct expression in ALI cells, of which two miRNAs, miR-16 and miR-502, were upregulated in primed EVs compared to naive EVs. In previous studies, miR-16 has been proven to promote alveolar fluid clearance and decrease the expression of TGF-β, IL-1β, and TNF-α, which all play a crucial role in lung injury (35–37). Our study further demonstrates the therapeutic importance of miR-16 in pulmonary disease, and suggests that it could be used as a prognostic biomarker for lung injury. The role of miR-502 in immune modulation remains uncertain; and to the best of our knowledge, no studies have investigated the role of miR-502 in lung diseases. However, several studies have identified miR-502 as an important tumor suppressor (38). Therefore, this miRNA may also have positive effects for respiratory disease treatment; however, further studies are required to verify these effects.

This study had some limitations. This study was conducted *in vitro*. According to previous studies, it has been reported that acute lung injury involves not only endothelial cells but also various immune cells, including neutrophils, lymphocytes, and macrophages, and studies on the therapeutic efficacy and complex mechanisms of primed EV through an *in vivo* ALI model is needed. Moreover, it is crucial to assess the potential side effects of primed EVs in an *in vivo* model. Pretreatment of stem cells with IFN-γ upregulates MHC class II expression, leading to antigen presentation to CD4^+^ T cells and immune activation (39, 40). Further research is necessary to ascertain whether the expression of MHC class II by stem cells affects EVs and whether immune activation occurs in animals injected with these EVs. The method used to identify EMT involved detecting markers that represented specific EMT stages. Therefore, no real-time method was used in this study, which is less sensitive for checking dynamic changes, such as EMT. Furthermore, in this study, ALI was induced by endotoxins. As many other causes of pulmonary diseases exist, the extrapolation of the results to other respiratory disease types is uncertain. Further studies are needed to determine the effect of the nine downregulated miRNAs, miR-184, miR-132, miR-130, miR-107, miR-1, let-7f, let-7g, let-7c, and miR-143, in lung inflammation. Although we found that primed EVs promoted lung endothelial cell proliferation, the specific mechanism underlying this phenomenon requires further study. Lastly, experiments were performed using a single dose of EV. Therefore, its capacity to repair lung damage in a dose-dependent manner is unknown. Additional research is needed on this as well.

Overall, we found that primed EVs had a better therapeutic effect in ALI *in vitro* model via suppressing TNF-α and IL-1β expression, while elevating IL-10 expression. Additionally, primed EVs decreased the level of TGF- β1 that induced EMT. This study suggests the potential of TNF-α and IFN-γ priming EVs from canine mesenchymal stem cells as a treatment for lung injury.

## Data availability statement

The datasets presented in this study can be found in online repositories. The names of the repository/repositories and accession number(s) can be found in the article/supplementary material.

## Ethics statement

The animal studies were approved by the Institutional Animal Care and Use Committee of Kangwon National University (KNU), Republic of Korea, and in compliance with the authorized guidelines (KNU, Protocol No. KW-230912-3). The studies were conducted in accordance with the local legislation and institutional requirements. Written informed consent was obtained from the owners for the participation of their animals in this study.

## Author contributions

J-SL: Writing – original draft, Investigation, Data curation, Conceptualization. Y-HJ: Writing – review & editing, Methodology. Y-HK: Writing – review & editing, Resources. J-HY: Writing – review & editing, Resources. J-OA: Writing – review & editing, Resources, Methodology. J-YC: Writing – review & editing, Resources, Methodology. J-HA: Writing – review & editing, Writing – original draft, Supervision, Resources, Methodology, Investigation, Funding acquisition, Conceptualization.

## Footnotes

^1^https://www.mirbase.org/
